# Anterior knee pain and femoral torsion in female patients: Rationale and outcomes of rotational femoral osteotomy

**DOI:** 10.1002/jeo2.70600

**Published:** 2026-01-08

**Authors:** Vicente Sanchis‐Alfonso, Robert A. Teitge

**Affiliations:** ^1^ Department of Orthopaedic Surgery Hospital Arnau de Vilanova Valencia Spain; ^2^ Department of Orthopaedic Surgery Wayne State University Detroit MI USA

**Keywords:** anterior knee pain, excessive internal femoral torsion, femoral anteversion, patellofemoral pain, rotational femoral osteotomy

## Abstract

Femoral and/or tibial torsional abnormalities are highly prevalent in female patients with anterior knee pain (AKP) who are recalcitrant to appropriate conservative treatment. In this paper, we focus on increased internal femoral torsion, which sometimes is an under‐recognised factor contributing to AKP. Two experts in patellofemoral pathology participated in conducting a review on the rationale and outcomes of rotational femoral osteotomy in female AKP patients and femoral maltorsion. Pathological femoral anteversion (FAV) is recognised as a cause of anterior knee pain. FAV is a problem because it changes the direction of the quadriceps and thereby the force acting on the patellofemoral joint. One of the key challenges in planning a rotational osteotomy is determining the precise degree of correction required. Murphy´s CT method is the closest to showing the anatomical reality when FAV is evaluated. Currently, there is no universally accepted cut‐off point at which rotational femoral osteotomy should be performed. Moreover, limited evidence exists regarding the optimal level at which the osteotomy must be done. In appropriately selected cases, intertrochanteric rotational femoral osteotomy represents a reliable treatment option for symptomatic excessive FAV, offering favourable outcomes with minimal complications.

**Level of Evidence:**

Level V.

**Abbreviations**
AKP: anterior knee pain
AV: acetabular version
FAV: femoral anteversion
IFT: internal femoral torsion
IRFO: intertrochanteric rotational femoral osteotomy
MPFLr: medial patellofemoral ligament reconstruction
TT: tibial tubercle
TTO: tibial tubercle osteotomy
TT‐TG: tibial tuberosity‐trochlea groove

## OVERVIEW

It is widely accepted that the vast majority of anterior knee pain (AKP) cases do not require surgery, and that symptoms may worsen following surgical intervention [24]. However, despite appropriate conservative treatment, only about 60% of patients report satisfactory outcomes [5]. This discrepancy raises an important question: could a subset of these patients be misclassified as candidates for conservative management? [24] It is likely that we are overlooking certain etiologies of AKP where surgery might actually be the most appropriate option [24]. One such aetiology is torsional deformity of the limb [24, 27, 29, 40, 41]. Sanchis‐Alfonso et al. [29] have shown that femoral and/or tibial torsional abnormalities are highly prevalent in female AKP patients who are recalcitrant to appropriate conservative treatment. While tibial and femoral torsional abnormalities were common (87% and 51% respectively), acetabular version (AV) abnormalities showed a markedly lower prevalence (17%), with deviations from the limit of normality no greater than 3° in most cases, suggesting that AV is likely not a relevant factor in the AKP patient [29]. Femoral maltorsion influences both patellofemoral and tibiofemoral contact pressure and therefore can be responsible for knee pain [17, 40].

In this paper, we focus on increased internal femoral torsion (IFT), which sometimes is an under‐recognised factor contributing to AKP [24]. In fact, some orthopaedic surgeons continue to conceptualise patellar malalignment – specifically tilt and/or lateral displacement – as isolated patella issue [23, 24]. In reality, patellar malalignment is frequently a consequence of abnormal femoral torsion, particularly excessive internal torsion [23, 24]. Therefore, a thorough evaluation of the femoral torsional profile is essential in all patients presenting with AKP [29].

Supporting this, Stroud et al. [38] followed a cohort of 92 children who, at age five, demonstrated a 30° greater internal hip rotation (in extension) than external rotation. At age 24, AKP was reported in 30% of those with increased IFT, compared to only 8% in the control group. Similarly, Winson et al. [43] found that 70% of adolescents undergoing arthroscopy for AKP exhibited increased IFT, a prevalence notably higher than the 33% observed in those treated arthroscopically for meniscal or cruciate injuries.

As early as 1995, Flandry and Hughston [8] emphasised that one of the most common causes of failure in extensor mechanism realignment surgery is the presence of unrecognised torsional abnormalities – namely increased IFT, excessive external tibial torsion, or both. These torsional issues, if not properly diagnosed, remain unaddressed and lead to poor surgical outcomes.

## WHY TORSION MATTERS: WHY DO WE PERFORM A ROTATIONAL FEMORAL OSTEOTOMY?

Many orthopaedic surgeons are primarily concerned with how to perform a rotational osteotomy, rather than when or why to do so. A revealing example of this gap in understanding is the common statement: “I am going to perform a femoral rotational osteotomy to correct femoral maltorsion and a tibial tubercle osteotomy (TTO) to correct the Q‐angle.” This suggests a fundamental misunderstanding of the biomechanical power of a rotational osteotomy, which in the authors’ opinion is significantly more effective than a TTO in correcting the Q‐angle [41]. The biomechanical rationale for this is that rotating the trochlea and the patella with it, should have a greater effect on the direction of the quadriceps force, compared to moving the tibial tubercle (TT).

### Why is excessive femoral anteversion a problem?

**Figure 1:**
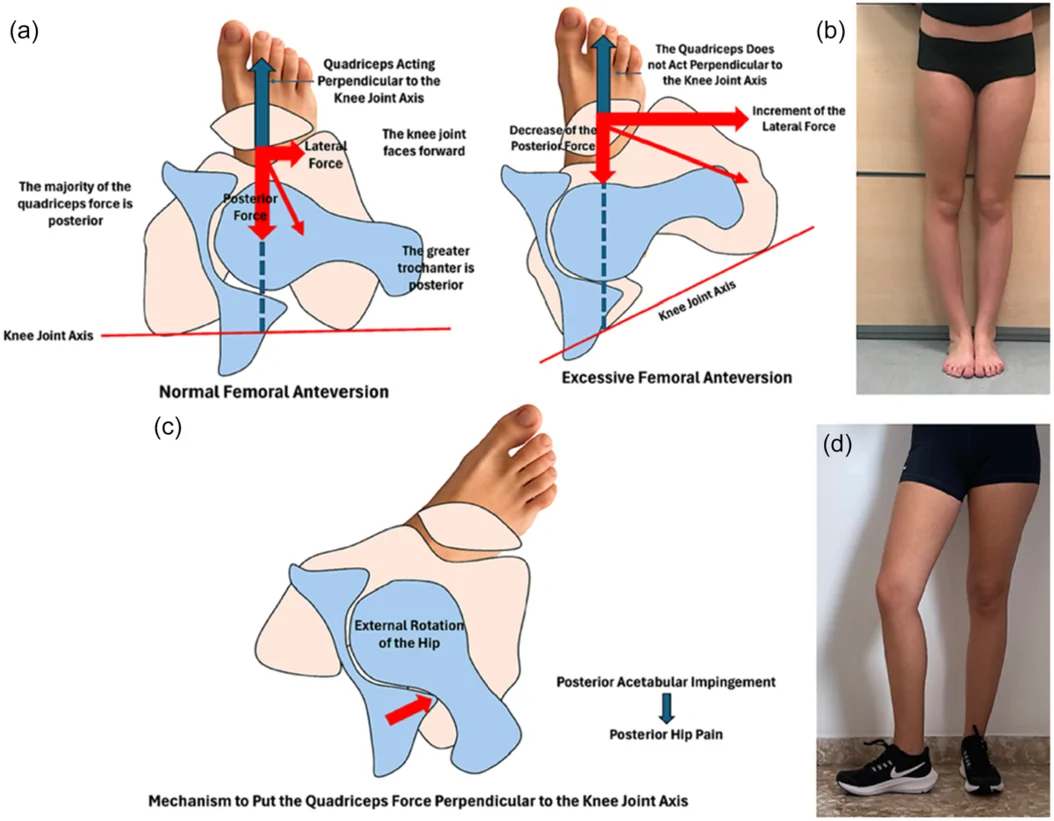
Illustration demonstrating (a) normal femoral anteversion (FAV), (b) excessive FAV and (c, d) the defence mechanism to avoid AKP (external limb rotation) (right knee). The máximum quadriceps efficiency is with knee joint facing forward in the direction the body is moving.

The quadriceps functions most efficiently when acting perpendicular to the knee joint axis, which lies in the distal femur and is likely parallel to the posterior condylar tangent (Figure 1a) [27]. The quadriceps plays a critical role in preventing the flexed knee from collapsing [27, 32, 41]. That is to say, the quadriceps is the muscle group that is primarily responsible for neutralising flexion torque in order to maintain upright stance on a limb when the knee is in flexion. It really does not fire when the knee is in the position of full extensión in the standing, weight‐bearing limb, because the centre of mass is anterior to the centre of rotation of the knee, resulting in a passive extensión moment [27]. The quadriceps force increases markedly as the knee is flexed, which is self‐evident if one performs squats of various depths in the standing position [27, 32, 41]. To stabilise the knee during flexion, total quadriceps force must increase, preserving the same ratio of posterior to lateral components, consequently the lateral force increases almost exponentially [4, 41]. In the presence of increased IFT, the quadriceps no longer acts perpendicular to the knee joint axis (Figure 1b) and therefore to control knee flexion, total quadriceps force must increase because the lateral component is increasing at the expense of the component that is actually producing extensión torque. This results in an increase in lateral force and a decrease in posterior force (Figure 1b). In other words, the quadriceps is less efficient and must exert greater overall force to produce a given amount of extension torque.

The only way to put the quadriceps force perpendicular to the knee joint axis is to rotate the joint axis back into the coronal plane. This might occur with external rotation of the hip, but that will cause posterior acetabular impingement which will limit the correction, and will put the hip muscles at mechanical disadvantage [27] and it will cause the foot to point outward (Figure 1c, d). These adversities can only be corrected with proper alignment of the hip joint axis, knee joint axis and ankle joint axis in the transverse plane.

Unfortunately, many orthopaedic surgeons focus on the damaged structures (cartilage and ligaments) rather than on the underlying cause of the damage – that is, the force responsible for the injury. Maltorsion leads to an abnormal distribution of force, both in terms of intensity and direction [17, 27]. If we focus only on the injured structure and not on the force that caused the injury, we are bound to fail [26].

### Why did we stop performing TTO in this patient cohort?

**Figure 2:**
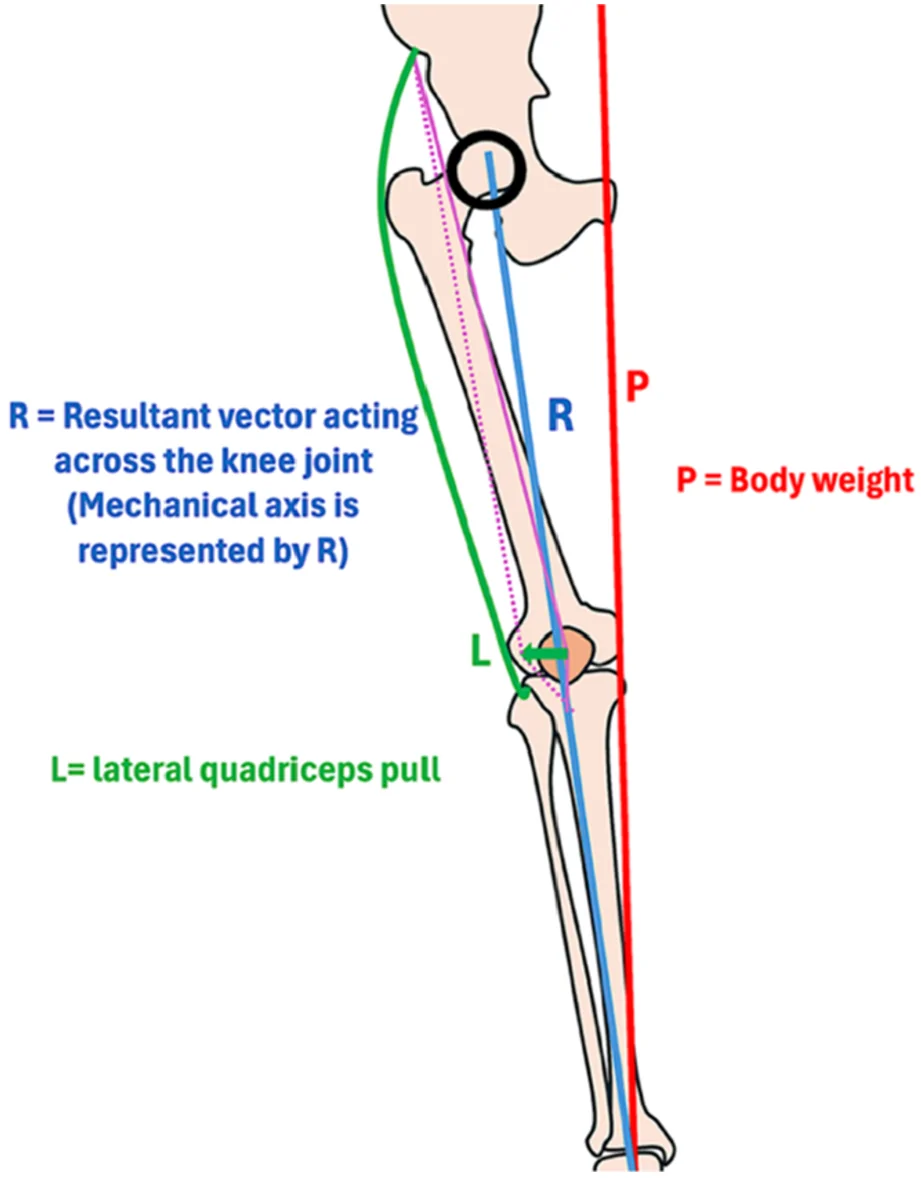
Illustration demonstrating forces across the knee joint.

The primary rationale for performing a TTO has traditionally been to alter the Q‐angle. The Q‐angle is the consequence of having a tibiofemoral valgus. The question arises: Why do we have a Q‐angle? Having a Q‐angle implies the presence of a small lateral force that is necessary for bipedal gait. Body weight is transferred from the body's centre of mass to the centre of the foot on the ground, a line that lies medial to the knee joint (Figure 2). This induces a varus thrust and medial overload at the knee. The lateral vector of the quadriceps offsets this effect, shifting the weight transfer to the centre of the joint and therefore the load is distributed evenly across the medial and lateral compartments (Figure 2) [15].

A TTO may be reasonable if the TT is truly lateralized. However, some studies have shown that in patients with chronic lateral patellar instability or AKP, the TT is not significantly lateralized compared to healthy individuals [29, 42]. Consequently, medializing the TT in such patients could disrupt the force balance, reduce the Q‐angle, and shift load medially. This can lead to overload of the medial tibiofemoral and patellofemoral compartments, increasing the risk of degenerative medial meniscal tears and medial compartment osteoarthritis [13, 19]. In such cases, TT medialization becomes iatrogenic. Moreover, in patients with excessive FAV, the knee points medially (Figure 1b), causing the entire patellofemoral joint to shift medially and thereby increasing the Q‐angle. Obviously, medializing the TT in these patients will not correct the malorientation of the knee [2].

At this point, it would be interesting to make some observations on the surgery of the TT in the patient with patellofemoral disease (lateral patellar instability) and IFT. Franciozi et al. [9] have seen diminished results from TTO combined with medial patellofemoral ligament reconstruction (MPFLr) in patients with recurrent patellar instability with increased FAV. In the same way, Zhang et al. [44] evaluated 144 consecutive patients with recurrent patellar instability. Patients were assigned into three groups: group A (FAV < 20°), group B (FAV 20°–30°), and group C (FAV > 30°). They have demonstrated that patients with FAV > 30° had inferior postoperative clinical outcomes and a higher rate of residual J‐sign after MPFLr combined with TTO. Thus, TTO does not prevent the negative effect of FAV on patellofemoral joint. Therefore, the best available evidence supports not performing TTO in patients with IFT.

### TT–TG distance: a flawed measurement

The tibial tubercle–trochlear groove (TT–TG) distance is often used as an indicator for TTO. However, this measurement is highly multifactorial and is more influenced by knee rotation than by true TT malposition [3, 11, 21, 35, 42]. Relying on TT–TG distance alone to guide surgical decisions is therefore misguided. We believe the method described by Tensho et al. [42] provides the most accurate assessment of TT location in the current literature. Using this approach, it has been demonstrated that TT is not lateralized in AKP female patients [30]. Thus, repositioning the TT appears unjustified in many cases. While moving the TT can indeed modify the Q‐angle, an increased Q‐angle is likely not caused by TT position. The TT–TG distance, therefore, is a flawed metric that fails to accurately describe true TT location.

## WHAT IS THE MOST EFFECTIVE METHOD TO ASSESS FEMORAL ANTEVERSION? WHAT IS THE THRESHOLD FOR SURGICAL INDICATION?

One of the key challenges in planning a rotational osteotomy is determining the precise degree of correction required. Several methods have been described for measuring femoral torsion, each with its own technical nuances and reference axes [12, 18, 34, 39]. Therefore, we should not talk about absolute values but about the method used to quantify femoral torsion. Kaiser et al. [12] have shown a significant difference in measurement techniques of even up to 11°, which can increase to 20° when people with exaggerated FAV are tested [33].

**Figure 3:**
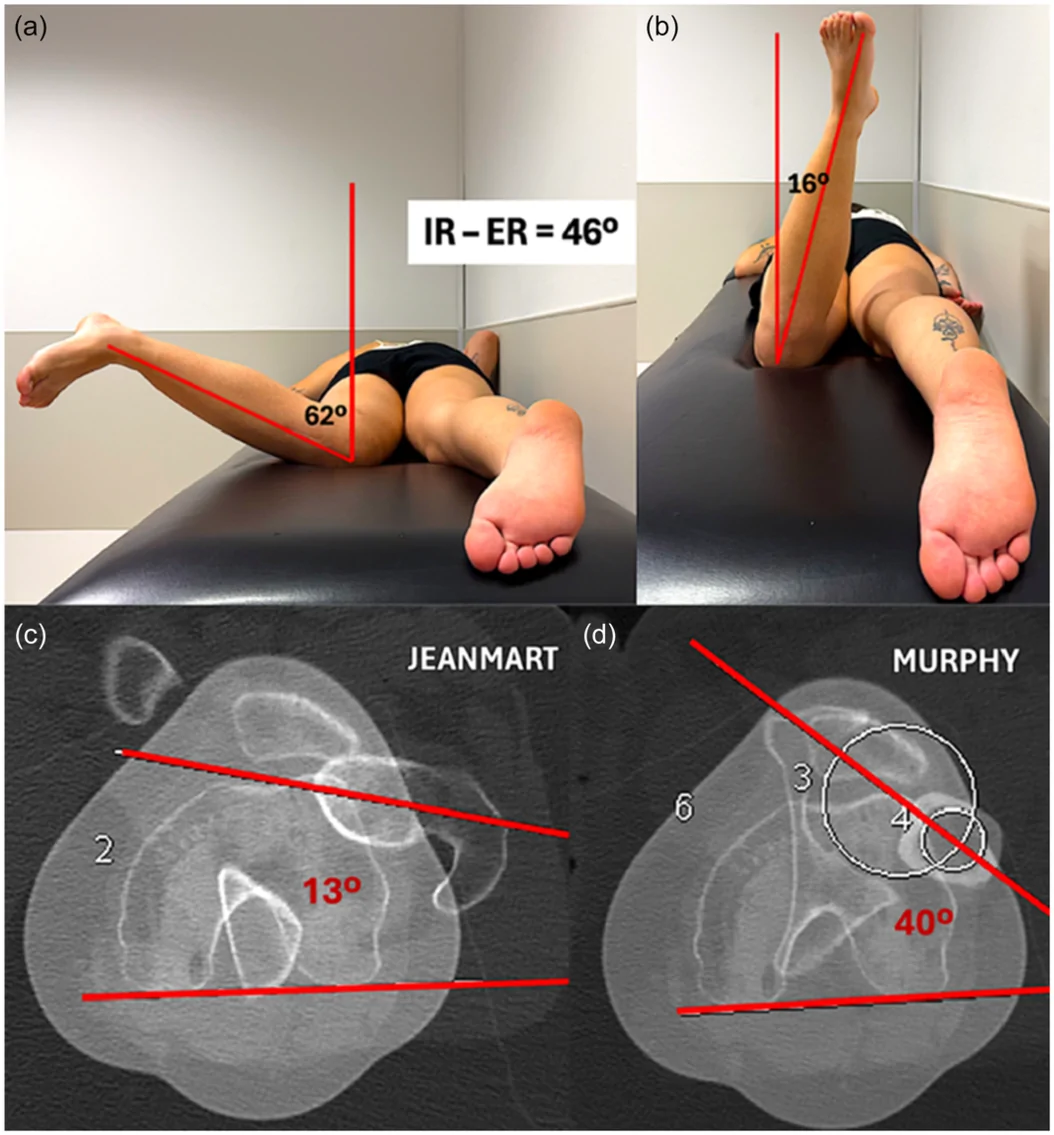
Left FAV. (a, b) Internal rotation of the left hip exceeds external rotation by more tan 30°. Measurement of FAV using the Jeanmart´s method (c) and the Murphy´s method (d). Murphy´s method reveals a value of 40° which is clearly pathological and coincides with what the physical examination reveals. However, according Jeanmart´s method FAV is completely normal.

In our clinical practice, we rely on the method described by Murphy et al. in 1987 [18]. We favour this technique because it closely reflects anatomical reality (Figure 3) and demonstrates good interobserver reproducibility. Compared with a semiautomatic neck‐fitting method [1], the method described by Murphy et al. [18] is the most accurate measurement and overestimates femoral version by only 3.5°. This overestimation is relatively small comparted with other CT‐based methods. Compared with the anatomic femoral version, the method described by Murphy et al. [18] overestimated femoral version by an average of 6.3° in a study comparing different measurement methods [39]. Moreover, Schmaranzer et al. [33] have observed that the differences between the classic method (the method that defines the proximal axis by a line along the femoral neck) and Murphy's method become more evident in patients with a clinical diagnosis of femoral torsional abnormality. Sanchis‐Alfonso et al. [28] have found significant differences between FAV values measured with the Murphy´s method and with the Jeanmart´s method in both the normal femoral torsion group (18.7 ± 5.52 vs. 14 ± 7.73; *p* = 0.003), and in the pathological FAV group (42.46 ± 6.45 vs. 28.1 ± 6.30, *p* < 0.001). The differences between both methods were higher in the pathological than in the normal control group (15.46, 95% CI 10.81–18‐07 vs. 4.67, 97% CI 1.73–7.86). What is more, at higher FAV magnitudes, the differences between the two methods were greater [28]. Finally, Murphy's method has shown very good interobserver reliability, ICC of 0.993 [7].

According to Meier et al. [16], the normal range of FAV using Murphy's method is 10° to 25°. Initially, we recommended rotational femoral osteotomy for patients with values exceeding 40°. However, as our clinical experience grew – particularly with female patients suffering from AKP – the surgical indication threshold was lowered to greater than 25°, in carefully selected cases. In the first author's series, rotational osteotomies were successfully performed in three patients with 26° of FAV, all of whom had severe, disabling pain without any other identifiable cause [31]. After extensive discussion with the patients and their families, surgical correction was pursued. The target postoperative anteversion angle was set at approximately 15°, meaning that in the case of a patient with 26°, a 10° correction was performed. Although 10° may seem minor, even small corrections in torsional abnormality can have substantial biomechanical effects and meaningful clinical impact (video). For example, in a patient with 40° of anteversion on the symptomatic side and 20° on the asymptomatic side, a 20° correction would be considered. The principle is to apply clinical judgement and common sense, tailoring the degree of correction to both the anatomical findings and the patient's symptoms.

## SURGERY

Although AKP is linked to torsional abnormalities, this does not mean that the presence of a torsional abnormality automatically leads to its surgical correction. We should always exhaust a tailored conservative treatment before osteotomy. There is no strong evidence base supporting specific torsion angle thresholds as indications for surgery [27]. However, based on the experience of both authors and their interpretation of the literature, a FAV greater than 25°, measured using Murphy's method, in the context of disabling AKP unresponsive to adequate conservative treatment, constitutes their current surgical threshold [31]. The primary indication for this surgery should be patients with pathological FAV causing severe functional limitations including gait disturbance and disabling pain (video). Rotational femoral osteotomy is contraindicated in the following situations: (1) AKP with FAV < 25° (Murphy's method); (2) Isolated FAV without clinical symptoms; and (3) requests for correction purely for cosmetic purposes.

### What is the ideal level for the osteotomy?

Correction of excessive FAV does not necessarily need to be performed at the distal femur. Generally, this location is often chosen simply because knee surgeons are less accustomed to operating on the proximal femur. From a mechanical standpoint, as long as the osteotomy is perpendicular to the long axis of the femur, the correction should be effective regardless of the level at which it is performed. Currently, no conclusive evidence supports a specific osteotomy level over another. For AKP caused by excessive FAV, the authors of this paper prefer an intertrochanteric rotational femoral osteotomy (IRFO).

In theory, osteotomies should be performed at the site of the deformity. Some have raised the question: where is the torsion? [28] The only answer is between the reference proximal transverse axis and the distal transverse axis. A third transverse axis would have to be defined to determine torsion above and below that level. Sanchis‐Alfonso et al. [28] performed research to determine whether the increased FAV in AKP females originates above the lesser trochanter, below the lesser trochanter or at both levels. They concluded that in chronic AKP female patients with increased FAV, the two segments of the femur contribute to the total FAV, with a different pattern among patients and controls, with the compensatory rotation of the diaphysis being much lower in pathological femora than in controls. In female AKP patients, excessive anteversion often results from a combination of increased proximal femoral neck anteversion and insufficient diaphyseal counter‐rotation (rotation of the diaphysis in the opposite direction) [28]. Performing the osteotomy at the level of the lesser trochanter may allow simultaneous correction of both abnormalities.

In cases of pathological FAV, the quadriceps line of action is no longer perpendicular to the knee joint axis, increasing lateral forces and reducing posterior force. This imbalance leads to greater patellofemoral shear stress and altered retinacular tension. A proximal osteotomy allows the quadriceps to use the full femoral length to gradually adapt to the new alignment.

### Outcomes

To date, only seven papers have reported on the outcomes of rotational femoral osteotomy for AKP in otherwise healthy young patients [6, 14, 20, 31, 36, 37, 40]. All reported favourable clinical results with low complication rates, though these studies were case series with small sample sizes and considerable heterogeneity regarding osteotomy level, fixation method, and outcome measures – making comparisons difficult.

In one publication, Robert Teitge references an unpublished review of 72 IRFO performed for AKP, with a mean follow‐up of 9.5 years [40]. He reported an improvement in the Kujala score from 39 to 89 and in the Tegner score from 2.2 to 4.0, with 95% of patients stating they would undergo the procedure again. The first author of this paper has reviewed 21 IRFO, all performed in young women with unilateral symptoms (mean age: 22 years; follow‐up: 1 to 6 years) [31]. In all cases: The only pathological finding was excessive FAV, none had patellofemoral arthrosis or patellofemoral instability; Knee MRI was normal, and TT–TG values were within normal limits. All patients had failed appropriate conservative treatment. None had prior knee surgery or associated hip pathology. Preoperative FAV averaged 38.2° ± 7.3° (range: 26°–53°), with a mean correction of 18.6° ± 4.9° (range: 10°–25°) [31]. The mean preoperative VAS score was 8.0 ± 1.3 (range: 4–10), improving to 0.4 ± 1.0 postoperatively (range: 0–4) [31]. The Kujala score improved from 45.3 ± 17.8 to 90.1 ± 6.4 (range: 84–100) [31]. Ninety‐three percent of patients indicated they would choose the same procedure again [31].

### Complications

In addition to general complications associated with osteotomy – such as nonunion, delayed union, infection, or neurovascular injury, all of which were rare [25] – some technique‐specific issues were observed. These included, trochanteric pain in 28% of patients, snapping hip in 9.5%, plate removal within 12–18 months in 20% of cases, and a non‐cosmetic prominence in the proximal thigh in 24% of cases [31]. Importantly, there were no major complications, such as deep infection, nonunion, or neurovascular injury. However, in patients with borderline femoroacetabular impingement, rotational correction may convert an asymptomatic cam lesion into a symptomatic one [10]. This highlights the importance of a thorough preoperative hip evaluation.

The types of complications seen in rotational osteotomy surgery are similar to those of the TTO. We have followed the same definition as Payne et al. [22] to compare the overall rate of major complications in IRFO vs TTO surgery. Major complications were defined as non‐union, fracture, infections and wound complications requiring return to the operating room, and deep vein thrombosis (DVT) or pulmonary embolism (PE). Payne et al. [22], in a systematic review, found an overall risk of major complications after TTO of 3.0%. In our series, the overall risk of major complications after IRFO was of 0% [31].

## ROTATIONAL OSTEOTOMY – A GAME CHANGER IN THE TREATMENT OF AKP

Chronic pain is a subjective phenomenon, and it is not easy to convince our colleagues that pain can be resolved by correcting an abnormal force distribution through rotational osteotomy. Moreover, the clinical tests used to evaluate a patient's pre‐ and postoperative condition are not sensitive enough to fully capture how influential torsional deformity can be in the genesis of symptoms – or how disabling maltorsion can become. However, there are cases, such as the one shown in the video, that allow us to affirm that IRFO is truly a game changer in the treatment of some AKP patients.

## CONCLUSION

Pathological FAV is often overlooked as a cause of AKP, yet it should be systematically assessed during the physical examination of every AKP patient. In appropriately selected cases, IRFO represents a reliable treatment option for symptomatic excessive FAV, offering favourable outcomes with minimal complications.

## NOMENCLATURE

Torsional pathology remains a controversial subject, beginning with the terminology itself [40]. In the specialised medical literature, the terms torsional abnormality and rotational deformity are often used interchangeably. However, the distinction between rotation and torsion is important. Rotation refers to the circular movement of an object around an axis [40]. In contrast, torsion describes a deformity that results from the twisting of a structure around its axis [40]. Rotating an object does not necessarily imply twisting it. For this reason, we prefer the term internal femoral torsion rather than internal femoral rotation, as the former more accurately reflects the pathological nature of the condition. That said, we find it appropriate to use terms like tibiofemoral rotation or knee rotation, which describe the angular relationship between the femur and tibia – specifically, the angle between a tangent to the posterior femoral condyles and a tangent to the posterior surface of the tibial plateau. Regarding the terms derotational vs rotational osteotomy, the prefix de‐ does not add any relevant information. Maybe, the prefix de‐ implies reduced rotation, but rotation can be either direction, it does not suggest which way or how much. Technically, it would be more accurate to refer to the procedure as a rotational osteotomy. Finally, the concept of version is defined as the angle between two axes located at opposite ends of a segment along the axis of rotation [40]. It reflects a situation where the proximal and distal axes are no longer parallel. However, the definition of proximal and distal axes can sometimes be subjective and lead to inconsistent interpretations.

## AUTHOR CONTRIBUTIONS

Vicente Sanchis‐Alfonso served as the project coordinator and was responsible for writing the manuscript. Robert A. Teitge reviewed the manuscript.

## CONFLICT OF INTEREST STATEMENT

The authors declare that they have no known competing financial interests or personal relationships that could have appeared to influence the work reported in this paper.

## ETHICS STATEMENT

Patients gave informed consent to the videos and agreed to treatment by written consent.

## Supporting information

Supporting information.

## Data Availability

None.
